# Cataract Surgery in Uveitis

**DOI:** 10.1155/2012/548453

**Published:** 2012-02-08

**Authors:** Rupesh Agrawal, Somashiela Murthy, Sudha K. Ganesh, Chee Soon Phaik, Virender Sangwan, Jyotimai Biswas

**Affiliations:** ^1^Tan Tock Seng Hospital, Singapore 308433; ^2^L. V. Prasad Eye Institute, Andhra Pradesh, Hyderabad 500034, India; ^3^Medical Research Foundation, 18 College Road, Tamil Nadu, Chennai 600006, India; ^4^Uveitis and Cataract, Singapore National Eye Centre, Singapore 168751

## Abstract

Cataract surgery in uveitic eyes is often challenging and can result in intraoperative and postoperative complications. Most uveitic patients enjoy good vision despite potentially sight-threatening complications, including cataract development. In those patients who develop cataracts, successful surgery stems from educated patient selection, careful surgical technique, and aggressive preoperative and postoperative control of inflammation. With improved understanding of the disease processes, pre- and perioperative control of inflammation, modern surgical techniques, availability of biocompatible intraocular lens material and design, surgical experience in performing complicated cataract surgeries, and efficient management of postoperative complications have led to much better outcome. Preoperative factors include proper patient selection and counseling and preoperative control of inflammation. Meticulous and careful cataract surgery in uveitic cataract is essential in optimizing the postoperative outcome. Management of postoperative complications, especially inflammation and glaucoma, earlier rather than later, has also contributed to improved outcomes. This manuscript is review of the existing literature and highlights the management pearls in tackling complicated cataract based on medline search of literature and experience of the authors.

## 1. Introduction

One of the most daunting tasks for an ophthalmic surgeon is the management of complicated cataracts. Cataract in uveitis may develop as a result of the intraocular inflammation per se, from chronic corticosteroid usage or more often from both [1]. The incidence of cataract in uveitis varies from 57% in pars planitis [2] to 78% in Fuchs heterochromic iridocyclitis (FHI) [3].

Cataract surgery in uveitic eyes is challenging and can present with many unforeseen intraoperative complications. Two decades ago, the outcome of surgery in these eyes was guarded and often confounded by postoperative complications such as severe inflammation, hypotony, and even phthisis bulbi. However, with modern day cataract surgical techniques, this is seldom the case. With improved understanding of disease processes, optimization of immunosuppression for perioperative control of inflammation, minimally invasive surgical techniques, availability of biocompatible intraocular lens material and design and surgeons trained in performing complicated cataract surgeries and anticipatory management of postoperative complications [4], the outcome has been maximized. Ocular morbidity in patients undergoing complicated cataract surgery is now limited to those cases that have pre-existing changes in the retina or optic nerve such as irreversible macular scarring or optic nerve atrophy. The fundamentals of cataract surgery in patients with different forms of uveitis have been recently reviewed by numerous authors [1, 4–17]. This paper presents a review of the existing literature on this topic and proposes a set of management pearls in tackling complicated cataract based on the literature review and authors' combined experience.

Extensive literature search using OVID medline search engine and all available library databases was employed with references cross-matching to obtain all peer reviewed articles published on cataract surgery in uveitis. The literature search included all relevant published studies on cataract surgery in uveitis since 1960 in English language.

## 2. Historical Perspective

Evolution of cataract surgery in uveitis. Until the advent of corticosteroids in the early 1960s, ocular inflammation was difficult and often impossible to control, and articles discussing the results of cataract extraction in inflamed eyes reported a high incidence of severe complications [4, 6, 7, 10].

In many cases, the complications resulted in marked reduction of vision or even loss of the eye [11]. More recent publications have reported a considerable decrease in the incidence of intraoperative and postoperative complications during cataract extraction in uveitis [4–7, 12]. The most likely reasons for this vast improvement would appear to be the increased ability to control inflammation perioperatively and the rapid evolution in microsurgical techniques that has occurred in recent years.

## 3. Review of Current Literature

### 3.1. Cataract Extraction in Different Uveitic Entities

Several factors must be kept in mind when assessing the current literature on cataract extraction in patients with uveitis. Inflammatory squealae may develop at relatively predictable rates in eyes with a given inflammatory syndrome [4–7, 11–17]. However, these rates do vary markedly among syndromes. It is therefore important to consider each syndrome separately when assessing the results and complications encountered following cataract extraction.

#### 3.1.1. Cataract Surgery in Patients with Fuchs Heterochromic Iridocyclitis (FHI)

By far the largest volume of information regarding cataract surgery in uveitis patients concerns those with FHI. The uveitis tends to be low grade and chronic, posterior synechiae rarely form, and patients are often unaware of the disorder until complications develop or the inflammation is discovered during a routine eye examination [18]. If patients are symptomatic, the two most common symptoms at the time of presentation are blurred vision as a result of cataract formation and vitreous floaters [18, 19]. The reported incidence of cataract formation in FHI syndrome ranges from 15% to 75%, with the vast majority of series reporting an incidence of around 50% [3, 4, 11, 13, 18, 19]. Most cataracts are of the posterior subcapsular type, with the remainder being cortical or mixed [18].

Numerous studies of cataract extraction in FHI have reported insignificant intraoperative and postoperative complications. To summarize, the recurring complications seen following cataract surgery in FHI, in order of reported frequency, were hyphema, progressive vitreous opacification, glaucoma, and spontaneously resolving vitreous hemorrhage. Other complications such as retinal detachment, extensive synechiae formation, and corneal edema were reported by some authors [3, 18, 19].

The reported incidence of intraoperative and postoperative hemorrhage varied from 3.6% to 76%. Recent articles report a much lower incidence of hyphema than was recorded previously, perhaps a result of the use of improved microsurgical techniques. Javadi and colleagues had safe outcomes with phacoemulsification and in-the-bag intraocular lens implantation in FHI, achieving a post operative visual acuity of 20/40 or better in all 41 eyes in their series. Vitreous haze was the major cause of postoperative visual acuity of less than 20/20. In the follow-up period of 17.8 ± 8.7 months, the only complication was PCO, which developed in six (14.6%) eyes [18]. The most visually significant complication of cataract extraction in FHI eyes appears to be the development of glaucoma. Permanent elevation of IOP was reported to develop at some time following the operation in 3% to 35% of these eyes [13]. The review of the literature reveals that, initially, control of intraocular pressure can be achieved by medication alone, but eventually up to 70% of these patients will require filtering surgery [13].

#### 3.1.2. Cataract Surgery in Patients with Juvenile Idiopathic Arthritis (JIA)

Not unlike inflammation associated with FHI, the chronic nongranulomatous anterior segment inflammation associated with this condition is often asymptomatic until complications supervene. However, the complications arising from this syndrome are more severe. The literature indicates that commonly encountered complications include band keratopathy, extensive posterior synechiae, and hypotony or glaucoma, in addition to cataract.

Rehabilitation of 40–60% eyes with JIA that develop cataract is considerably more difficult than that in eyes with FHI [20–31]. Numerous studies have reported the poor post operative outcome of conventional cataract extraction even without intraocular lens implantation. The largest series of patients with JIA who had cataract extraction was reported by Kanski and Shun-Shin [32]. Out of 162 eyes, 61 had the cataract removed by needling and aspiration, and 101 had lensectomy and limited anterior vitrectomy. Vision was hand motion or less in l5% of the lensectomized eyes, 20/400 to count fingers in 30%, and better than 20/60 in 56%.

It was not until the important concept of adequate immunosuppression aimed at zero tolerance of inflammation and abolishment of every cell was strongly advocated and supported by clinical studies [22, 33] that patients with JIA undergoing cataract surgery saw better visual outcomes. In his landmark paper, Foster described the intensive use of preoperative and postoperative corticosteroids and reported visual acuities of 20/40 or better in 67% of patients and no major intraoperative or postoperative complications [22]. Subsequently several studies reported successful postoperative outcome with limited complications with perioperative and postoperative immunosuppression. Treatment with systemic, topical, and periocular steroids is recommended during the perioperative period for all eyes with uveitis associated with JIA that undergo cataract extraction. Surgery should be delayed until the anterior chamber is free of inflammatory cells (“flare” will persist).

The addition of a limited vitrectomy in combination with lens removal resulted in a decrease in the incidence of phthisis from 25% to 2% in a series described by Kanski [34]. Another similar study revealed the beneficial outcome with vitrectomy in patients with JIA-associated uveitic cataract [35].

Successful use of intraocular lens implantation in uveitis was reported by Probst and Holland in 1996 wherein visual acuity of 20/40 or better was achieved in eight eyes at an average follow-up period of 17.5 months; however the study was limited by very small number of patients [26]. The patients were operated at around a mean age of twelve years, which was almost five years after the diagnosis of JIA in this study as against the midteens in other studies.

A more recent study by Kotaniemi and Penttilä in 2006 reported good postoperative outcome following intraocular lens implantation in patients with juvenile idiopathic arthritis-associated uveitis where cataract surgery with intraocular lens implantation was performed in 36 eyes and the mean postoperative follow-up period was 3.3 years. The visual result was good (>0.5) in 64%, moderate (0.3–0.5) in 11%, and poor (0.3) in 25% eyes. Secondary cataract developed in 16 eyes but in none of the eyes with primary posterior capsulotomy and anterior vitrectomy. Secondary glaucoma developed in 18 eyes, retinal detachment in 2 eyes, cystoid macular edema in 16 eyes, and band keratopathy in 12 eyes [36].

Another major study published in 2009 by Quiñones et al. looking at cataract extraction in children with chronic uveitis with 21 out of 34 children having JIA reported good tolerance of intraocular lens in JIA patients and good postoperative visual outcome with optimal control of inflammation with immunomodulatory therapy. The average followup reported in this study was more than four years [33].

Recently published study by Ganesh and colleagues analysed ten eyes of 7 patients who had phacoemulsification with IOL implantation done by a single surgeon. A heparin surface modified IOL was used in 7 eyes and a foldable acrylic IOL was used in 3 eyes. At final followup, 70% of eyes had a visual acuity of 20/40 or better and 30% had improved visual acuity to 20/60. Posterior capsular opacification was found in 2 eyes and anterior capsular fibrosis in 1 eye [37].

Most JIA patients are in the amblyogenic age when they develop cataracts and the surgeon must bear this in mind when planning cataract surgery. For this reason, cataract surgery cannot be withheld or delayed for too long whilst battling to keep the eye quiescent in preparation for the operation. Furthermore, primary capsulotomy with limited anterior vitrectomy may be considered in children under the age of six to eight years as performing YAG capsulotomy in the postoperative period for posterior capsular opacification may be difficult in a very young child.

A number of factors combine to make cataract surgery more hazardous in these patients. These eyes have a marked tendency to form synechiae [22]. Implantation of an IOL into the capsular bag at the time of cataract removal often results in reformation of posterior synechiae and development of membranes over the IOL, resulting in cocooning of the IOL [26]. This may results in malignant glaucoma if the iris is plastered back onto the anterior capsule. If seclusion or occlusion pupillae occur, secondary pupil block glaucoma develops warranting a surgical peripheral iridectomy [26]. With cyclitic membrane formation, traction of the ciliary processes and ciliary body result in hypotony and phthisis bulbi if surgery is not done early. Although glaucoma is common in this syndrome due to the chronicity of inflammation, with a reported incidence of about 25% in most studies, some eyes are hypotonous by the time cataract extraction is contemplated [22, 35]. In addition to making surgery technically more difficult, hypotony is associated with an increased risk of postoperative choroidal effusion, macular edema, and phthisis. As a result of the high risk of complications that may develop following surgery, there is still controversy surrounding the implantation of an IOL at the time of surgery [29]. These complications are still very real despite the development of biocompatible IOL materials. Thus, even with successful cataract surgery, the outcome of cataract surgery in JIA patients is often limited by the state of the optic nerve and the macula and may be compromised by the presence of band keratopathy [21, 22, 24].

#### 3.1.3. Cataract Surgery in Patients with Intermediate Uveitis

Inflammation in eyes with pars planitis is limited primarily to the posterior segment, although mild anterior chamber activity is present in some cases. This contrasts markedly with the inflammation seen in Fuchs syndrome or JIA-associated uveitis, which is located primarily anterior to the lens. As the anterior chamber is largely free of inflammation in intermediate uveitis, synechiae seldom develop and the incidence of glaucoma is low [2]. As chronic inflammation persists in close proximity to the lens, cataracts eventually develop in 40% of patients [2]. Lens opacities first develop as a diffuse haze in the posterior subcapsular region. A large percentage of the cataracts remain at this stage; only half of the cataracts in one large series eventually became visually significant [2].

Macular edema is the major complication encountered following surgery in pars planitis patients and is the most important cause of poor vision. It has been seen to some degree in almost half of the eyes that undergo cataract extraction and is responsible for 80% of the eyes with less than 20/40 vision. Generally, few other complications are seen, and inflammation appears to remain under control following surgery. The incidence of glaucoma after cataract surgery in uveitis averages around 10%, which closely parallels the natural rate of glaucoma in this syndrome [7]. Numerous studies have reported varying results of cataract extraction in patients with intermediate uveitis [2, 4, 20, 38–42]. A possible reason for the varied outcomes is that intermediate uveitis can take on a variable clinical course, with approximately a third of all patients having a severe prognosis despite therapy. There have been a few studies which showed good postoperative outcome with vitrectomy along with cataract extraction in patients with chronic intermediate uveitis [43–47].

A large study by Ganesh and colleagues in 2004 [38] analyzed the outcome of phacoemulsification with intraocular lens implantation in 100 eyes with intermediate uveitis. In this study, 91% of eyes showed a favorable visual outcome at average followup of 19.67 months. The major complications reported by authors in this study were significant posterior capsular opacification which occurred in 10%, CME in 50%, reactivation of intermediate uveitis in 51%, IOL deposits in 29%, IOL decentration in 1%, and anterior capsule fibrosis in 14%. The three most frequent causes of poor visual recovery were CME, submacular fibrosis, and epiretinal membrane. The authors concluded that phacoemulsification with IOL implantation in eyes with pars planitis was safe and led to good visual outcomes in most cases. They attributed the success to control of inflammation, meticulous surgery, in-the-bag IOL implantation, and vigilant postoperative care.

#### 3.1.4. Cataract Surgery in Behcet's Disease

Cataract formation is the most common anterior segment complication after recurrent inflammation, occurring in up to 36% of cases [1]. It was reported that the postoperative visual acuity was found to be significantly lower in eyes with BD than in those with idiopathic uveitis because of the severe posterior segment complications, mainly optic atrophy [48].

Surgery is indicated whenever visual improvement can be expected and the eye has been free of inflammation for a minimum of 3 months. Operating on eyes with cataract and uveitis has been previously reviewed by Foster and associates [8]. Their recommendations for a successful cataract surgery and for minimizing the postoperative uveitis are as follows.

Uveitis should be inactive for at least 3 months preoperatively, systemic and topical steroids should be used prophylactically for 1 week preoperatively and continued postoperatively, immunosuppressive drugs should be continued, complete removal of cortical material should take place, and one-piece PMMA posterior chamber intraocular lens should be used if the patient and the surgeon understand the special nature of this surgery, its risks, and the prognosis for success.

In another paper by Berker et al., the authors reported results of phacoemulsification and intraocular lens implantation in patients with Behcet's disease [49]. They reported 72.5% of eyes had improvement in vision after surgery. However, the vision got worse in 17.5% of the eyes. Most frequent complication reported by them was posterior capsular opacification in 37.5% of eyes. Other complications were posterior seynchiae and severe inflammation. Posterior segment complications such as epiretinal membrane formation, cystoid macular edema, and optic atrophy were also reported by the authors.

#### 3.1.5. Cataract Surgery in Patients with Idiopathic and Other Forms of Uveitis

This group includes patients with uveitis associated with sarcoidosis, toxoplasmosis, Vogt-Koyanagi-Harada (VKH) syndrome, sympathetic ophthalmia, and other types of uveitis. Duke-Elder [50] and Smith and Nozik [51] both reported based on anecdotal evidence that such patients do well following conventional surgery, as long as inflammation has been absent for at least two to three months preoperatively. Moorthy et al. [52] performed cataract surgery in 19 eyes of VKH. 68% of eyes had best corrected visual acuity (BCVA) of >6/12. The most common reason for BCVA <6/12 was pigmentary disturbance in macula. In 1983, Reynard and Meckler [53] reported the results of cataract extraction in six sympathizing eyes of patients with sympathetic ophthalmia. All eyes showed minimal inflammation at the time of surgery, two eyes underwent intracapsular cataract extraction, three extracapsular cataract extraction, and in one case the cataract was needled. Following surgery, all the eyes required steroid treatment during followup to control recurrences of inflammation. Uncontrolled inflammation led to the formation of cyclitic membranes or phthisis in three eyes in spite of corticosteroid therapy. Two eyes achieved visual acuity better than 20/40 during the follow-up period, which ranged from one to 23 years. The three eyes with severe postoperative inflammation retained only light perception vision; one eye, with chronic inflammation and macular edema, retained 20/100 vision.

Akova and Foster [54] analyzed results in 21 eyes of sarcoidosis. 61% eyes achieved a stable visual acuity of >6/12. In 2004 Ganesh et al. [55] reported results of cataract surgery in 59 eyes of VKH and found BCVA improvement by one or more lines on Snellen's chart in 40 (67.79%) eyes. PCO was seen in 38 (76%) eyes, followed by optic atrophy and subretinal gliosis.

Fox et al. [23] described 16 patients with various types of uveitis associated with ankylosing spondylitis in 5 and inflammatory bowel disease in two. All patients had less than 0–2 anterior chamber cells for at least three months preceding surgery. Cataracts were removed by extracapsular techniques, including phacoemulsification, and 14/16 eyes had posterior chamber intraocular lenses implanted. Vision improved in all cases, with most eyes achieving 20/40 or better visual acuity. Few complications were noted, the most serious appeared to be the development of posterior synechiae and in 6/14 eyes (43%), macular pathology was seen postoperatively.

## 4. Current Guidelines for the Management of Uveitic Cataract

### 4.1. Clinical Evaluation of Complicated Cataract and Associated Uveitis

The eye in which visual loss is mainly attributable to cataract formation is most likely to benefit from cataract surgery. The outcome of surgery depends upon several factors, namely the uveitic diagnosis, proper perioperative management and meticulous surgery. The specific uveitic diagnosis is of paramount importance also when planning the surgical strategy [4], such as determining whether an intraocular lens should be implanted or not.

Diseases that spare the posterior segment generally have a better prognosis than those that affect the macular and/or optic nerve. Acute uveitic syndromes tend to be associated with better outcomes than chronic uveitis. Thus, JIA patients, especially those with anterior uveitis in the pediatric age group [56] have more poor outcome than patients with ankylosing spondylitis and anterior uveitis.

**Figure 1:**
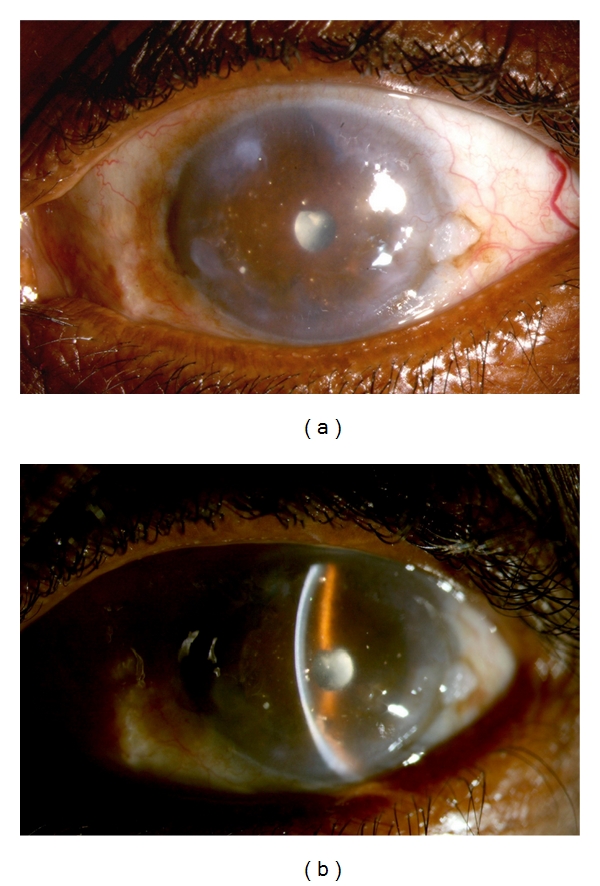
A 54-year-old farmer gave history of loss of vision and repeated episodes of redness and pain in the left eye following injury 40 years ago. B scan of the left eye showed findings suggestive of old vitreous hemorrhage and retinal detachment. Since visual acuity was doubtful perception of light, surgery was not advised for him. Diffuse (a) and slit (b) photographs show the left eye with peripheral corneal scar and peripheral anterior synechiae and total posterior synechia with cataract. At the temporal periphery, there is an incidental conjunctival lesion suggestive of actinic keratosis. Note the polychromatic crystals (cholesterolosis) deposited on the iris.

The visual potential of the eye, determined by prior permanent structure damage, should be carefully determined before planning cataract surgery as this will have a direct impact on the visual outcome. The state of the macula and optic nerve should be thoroughly examined for during the preoperative assessment. Macular ischemia, atrophy, chronic macular edema, or scar, such as that resulting from a choroidal neovascular membrane, are poor prognostic factors. Similarly, optic atrophy and severe cupping of the optic disc are bad prognostic signs. Furthermore, the state of the retina must also be carefully examined for evidence of ischemia. In presence of dense lens opacity, B scan ultrasonography should be done to rule out retinal detachment which may complicate the eye with uveitis. In eyes with chronic retinal complications, possibly the cataract surgery may not result in optimal and desirable visual outcome and such cases are left to the discretion of surgeon to operate under nil visual prognosis or for cosmetic reasons (Figures 1(a) and 1(b)). Less disabling abnormalities such as pre-existing corneal scars and severe iris atrophy may also compromise the visual outcome.

Regardless of the guarded prognosis, a definite indication for cataract removal in an eye that is not blind, is phacoantigenic uveitis. This may result from the hypermature state of a cataract, whereby lens proteins leak out of the capsular bag through an intact capsule, or in cases of trauma, where the lens capsule has been breached, leading to persistence of intraocular inflammation. Cataract surgery may also be indicated to permit better visualization of the posterior segment for appropriate medical or surgical management of the eye [57].

### 4.2. Control of Pre Operative Inflammation

**Figure 2:**
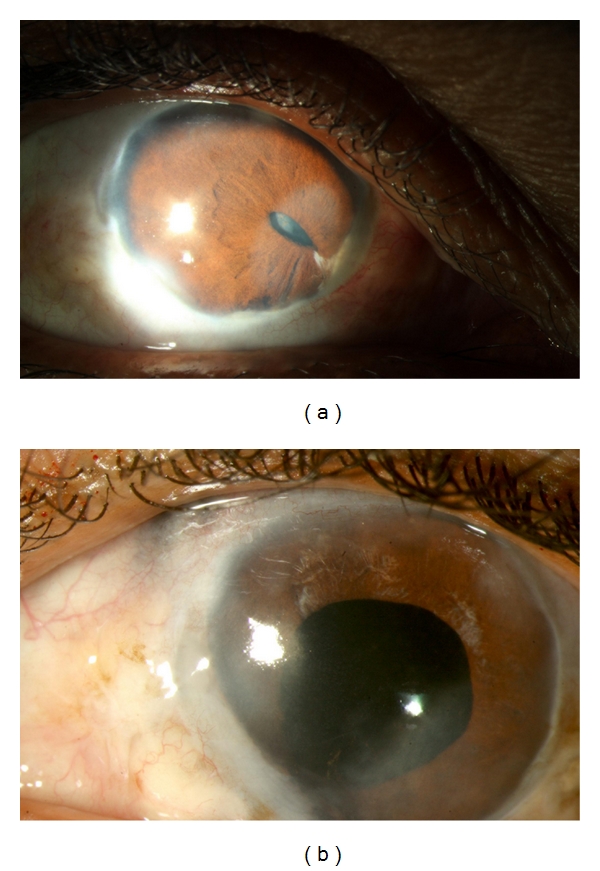
A 48-year-old lady was diagnosed as bilateral sclera-keratouveitis, with complicated cataract. She was investigated extensively and had a positive Mantoux test, for which she received anti-tubercular therapy. She underwent cataract surgery in the left eye with a preoperative visual acuity of counting fingers. Postoperatively, her visual acuity improved to 20/125, limited by the presence of a central corneal scar. (a) Shows the right eye showing evidence of healed scleritis, corectopia, and a complicated cataract. (b) Shows the left eye two months postoperatively.

The risk of reactivation of uveitis must be assessed. Jancevski and Foster recommended the use of supplementary perioperative anti-inflammatory therapy to prevent damage to ocular structures essential to good vision [58]. This has been shown to reduce the risk of postoperative CME [59]. In eyes which are at risk of developing macular edema postoperatively, such as chronic anterior uveitis secondary to sarcoidosis; or eyes with previous episodes of CME (e.g., intermediate uveitis), steroid prophylaxis should be given perioperatively to protect against recurrence of macular swelling. Similarly, steroid prophylaxis should be administered in eyes at risk of developing recurrence of uveitis following cataract surgery, for example, Vogt-Koyanagi Harada disease, Behcet's disease and birdshot choroidopathy, to name a few. This may take the form of oral steroids 1 mg per kg/day starting 3 days preoperatively, tapering the steroid dose according to the amount of inflammation postoperatively. Generally, the oral steroids are tapered or reduced to the preoperative levels over the subsequent month, whilst maintaining the dose of other concurrent immunosuppressive therapy. Alternatively, if there are no contraindications to periocular steroid injections, such as documented steroid response or infectious uveitis, an orbital floor or sub-tenon's injection of depot steroid, such as triamcinolone acetonide 40 mg/1 mL may be given, especially in patients where high doses of oral steroid are contraindicated, for example in poorly controlled diabetics. In addition, guttae prednisolone acetate 1% 2 hourly administered 2 days prior to surgery, together with an oral and topical non-steroidal anti-inflammatory agent, can be given. Supported by encouraging results in the recent literature, the authors favour an intravitreal injection of preservative-free triamcinolone acetonide 4 mg in 0.1 mL at the conclusion of cataract surgery [60–62]. This has been shown to be as effective as prescribing systemic steroids perioperatively. Similarly, optimal control of periocular inflammation is imperative in cases with sclerokeratouveitis for optimal surgical and visual outcome (Figures 2(a) and 2(b)). In eyes with types of infectious uveitis that have a propensity to recur, such as ocular toxoplasmosis and herpes simplex uveitis, preoperative prophylaxis should also be considered as surgery may trigger reactivation of the infection. Toxoplasmic retinochoroiditis is associated with a 36% risk of reactivation following surgery [63]. Herpes simplex is also associated with reactivation following the stress of surgery, and acyclovir 400 mg bid or valtrex 0.5 g qd preoperatively and for 2 to 3 weeks postoperatively may help prevent recurrence. In addition, topical NSAID and prednisolone acetate 1% or even oral NSAID may help control postoperative inflammation [64].

### 4.3. Complications of Uveitis Adversely Affecting Surgical Outcome: High-Risk Surgical Cases

Determining the surgical risk is a very important aspect of preoperative assessment. Eyes which have generally low intraocular pressures, especially readings of 6 mmHg or less even when quiescent, are at high risk of developing postoperative hypotony or even phthisis bulbi. Other warning signs such as seclusio papillae with normal intraocular pressure reading and apparent phacodonesis without evident zonulysis are important poor prognostic signs for postoperative hypotony. Eyes in which the uveitis is difficult to control are also at high risk of severe postoperative inflammation and hypotony or phthisis bulbi. The presence of choroidal effusion on B scan ultrasonography or diffusely thickened choroid is a poor prognostic sign. Conducting a careful ultrasonic biomicroscopy is essential in eyes with relative hypotony [65] to assess the state of the ciliary body and its processes. If the ciliary body has undergone atrophy, the risk of hypotony is high. If the ciliary body is found to be detached and processes appear under traction from a ciliary (cyclitic) membrane, cataract surgery should be combined with vitrectomy and trimming of the ciliary membrane aided by indentation of sclera to relieve ciliary body traction and to restore normal IOP.

### 4.4. Diagnostic Aids

Apart from the standard means of assessing macular, optic nerve and retinal function, one may apply additional methods such as pupillary response, light projection, colour perception and B scan ultrasonography, or an attempted OCT in looking for macular atrophy, edema, or hole [66]. Performing a macular potential test using laser interferometry may help in determining minimum visual potential [67]. A fundus fluorescein angiogram may also demonstrate macular ischemia or edema, retinal ischemia, active posterior segment disease, including disc leakage [68]. Considering the risk of post operative hypotony and to rule out cyclitic membrane preoperatively in chronic uveitic cases with bound pupils as described above, ultrasound biomicroscopy can also aid preoperative surgical planning [65]. Finally, the laser flare meter is a useful tool to measure flare in the anterior chamber and may be used to determine if the eye is quiet. It also helps guide therapy as it can be used to monitor the level of inflammation in the anterior chamber during the postoperative period [69].

### 4.5. Optimal Time for Cataract Surgery

Before scheduling surgery, the ophthalmologist should attempt to ensure that the eye has been quiescent for at least 3 months [11]. This has been shown to reduce the risk of postoperative CME [59]. In cases where, despite heavy immunosuppression, the intraocular inflammation is still not completely abolished and surgery is urgently required, such as in an intumescent cataract, the patient may be administered intravenous methylprednisolone 1 g one day before surgery. A study from Japan suggests that in patients with Behcet's disease the eye should be inactive for a minimum of 6 months and that the risk is higher if attacks have occurred within 12 months of cataract surgery [70].

### 4.6. Counselling of the Patient for Uveitic Cataract Surgery

The most important aspect of counselling when planning to perform cataract surgery for the uveitic eye is explaining the visual prognosis. The general risks involved in surgery, such as infection and other intraoperative complications will also need to be thoroughly explained, especially if there is phacodonesis, hypotony, or glaucoma. Emphasizing that the eye will need a minimum period of quiescence before surgery to minimise the chance of recurrence and improve the visual outcome is important. Furthermore, it is important to explain that surgery may be complicated and possibly take longer than usual because of abnormal anatomy, such as the presence of synechiae, membranes, and so forth, and that these factors may contribute to postoperative inflammation. Other factors requiring careful discussion and explanation include the possibility of and reasons for delayed visual recovery, the need for compliance with medications (systemic immunosuppression may need to be adjusted), and frequent followup, especially if the patient has difficulty accessing medical care.

These patients are often young, therefore losing a lens that was still able to accommodate in exchange for an intraocular lens implant means loss of accommodation. Consequently, they will need to understand and accept the fact that they will now need reading glasses. The type of IOL implant, the material, and design are all important points that need discussion. The choice of intraocular lens can be based on the extensive literature available [39, 71–74].

Generally, multifocal implants, whether based on diffractive or refractive principles may compromise the visual outcomes due to the presence of preexisting macular or optic nerve conditions. Hazy or scarred vitreous gel contributes to poor contrast sensitivity and any previous episode of inflammation with macular involvement increases the risk of poor visual performance with multifocal implants. These patients do better with a monofocal IOL implant. Even accommodative IOLs may not be effective in the long term due to recurrent inflammation and scarring of the ciliary body, or slowly progressive fibrosis of the capsular bag.

Posterior capsule opacification is another frequent complication encountered postoperatively because of the relative youth of these patients [75]. Choice of IOL, as will be discussed later (see IOL implantation-contraindications and type of IOL), and surgical technique are major determining factors as well. Occasionally, opacities are observed during the surgery, and some surgeons prefer to perform a primary posterior capsulorhexis at the time of the cataract surgery before implanting the IOL [76]. This possibility should be discussed with the patient before surgery, especially in view of the increased risk of postoperative endophthalmitis, CME, and retinal detachment.

Should the patient have complications other than cataract formation alone, the option of separate, staged, or combined surgery should be discussed with the patient and advice given regarding their risks and benefits [77].

## 5. Surgical Technique

### 5.1. Choice of Surgery

The choice of cataract surgery technique is best left to the surgeon and depends upon the individual surgeon's surgical skill and experience. Cataract removal by phacoemulsification is safer for the uveitic cataract as less inflammation is induced than that by a manual extracapsular cataract extraction. During the surgery, the anatomy of the anterior segment should be restored to a state as close to normal as possible.

Some uveitic cataract eyes are complicated by glaucoma or retinal problems that may also benefit from surgery. For eyes with concomitant uveitic glaucoma, surgery is preferably not combined with the cataract surgery as the risk of bleb failure is increased with drainage of post-cataract surgery inflammatory exudate through a healing bleb. Where possible, cataract surgery should be done first. Regarding retinal complications, such as epiretinal membranes or coexisting retinal detachment, cataract surgery may be combined with vitreoretinal surgery. In cases with major retinal problems, the eye may be safely rendered aphakic until the retinal problem has been dealt with. In eyes with intermediate uveitis, or FHI, cataract surgery may be combined with vitrectomy, performed to clear the vitreous gel, thereby reducing vitreous clouding. In intermediate uveitis, this often not only improves vision but also controls intraocular inflammation and helps resolve the cystoid macular edema. At the end of surgery, especially with combined surgical procedures, having excluded steroid responders and eyes with infectious uveitis, an intravitreal injection of triamcinolone acetonide, is often adequate to control the postoperative inflammation and prevent CME. The risks and benefits of combining or separating the surgical procedures should be thoroughly explained to the patient.

### 5.2. Intraoperative Surgical Techniques and Skills [78]

**(a) Posture**

Patients with ankylosing spondylitis with a fixed flexion deformity of the axial spine, especially when the cervical spine is involved, have difficulty not only in placing their chin on the slit-lamp rest but also lying flat on the operating table for ocular surgery. These patients are best postured in the Trendelenburg position, whereby their lower limbs are elevated above the level of their head, so as to maintain the plane of the face parallel to the floor. The pillow support may need to be stacked up high in order to support the head. As the patient tends to slide down the bed, a strap is best secured around the torso to prevent the body from slipping.

**(b) Surgical Challenges**

The uveitic eye poses numerous surgical challenges. These include the small pupil, shallow anterior chamber, posterior synechiae, peripheral anterior synechiae, pupillary membranes and even zonulolysis. Complications that may arise from the problems include an undersized or incomplete capsulorhexis, iris prolapse, increased risk of posterior capsular rent, increased risk of intraoperative zonular dehiscence, and increased postoperative inflammation.

**(c) Anaesthesia**

Whilst phacoemulsification surgery may be done under topical anesthesia, manipulation of the iris may induce ocular discomfort or pain. Either regional anesthesia or an intracameral injection of preservative-free lignocaine 1% can provide adequate analgesia. For children and in patients for whom prolonged surgical time is anticipated, as in severe zonulolysis requiring modified capsular tension rings that need suturing, general anesthesia may be preferred.

**(d) Incision**

Either a scleral or temporal clear corneal incision may be used. However, the incision should be of adequate length in order to prevent iris prolapse in eyes with small or stretched pupils.

**(e) Pupil Enlargement**

An attempt at pupil dilation can be made by injecting balanced salt solution with adrenaline (1 : 1000 0.5 mL adrenaline in 500 mL) into eyes with pupils that are not bound by synechiae or membranes. Preservative-free intracameral lignocaine 1% may also be used to help dilate the pupil only if it is not bound. Choosing a viscoadaptive viscoelastic such as Healon 5 (sodium hyaluronate 2.3%, Abbott Medical Optics) is useful as this high-molecular-weight sodium hyaluronate can physically roll open the pupil and keep it dilated as long as the aspiration flow rate is kept low.

**(f) Synechiolysis and Removal of Pupillary Membrane**

**Figure 3:**
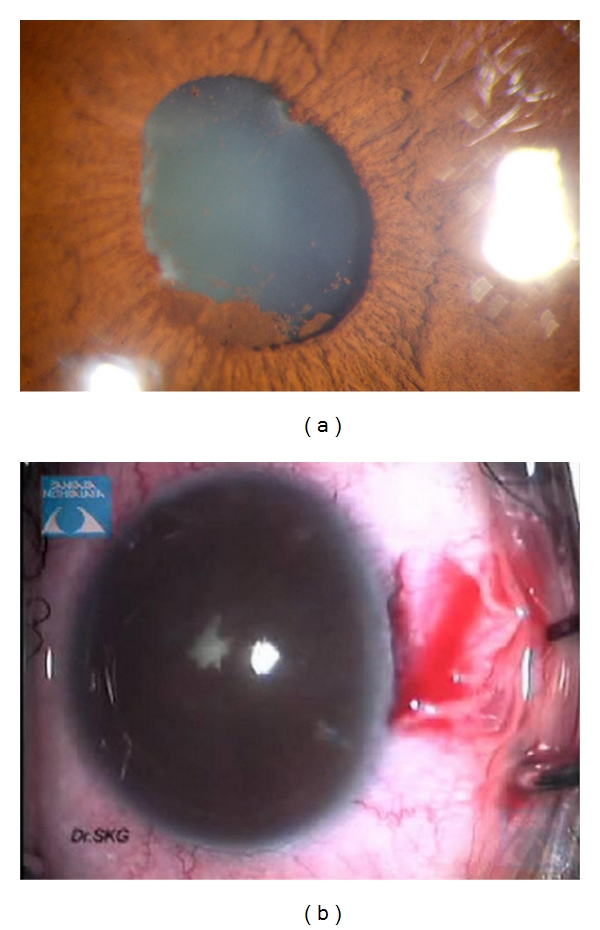
(a) Showing the eye with synechiae at papillary border, pigment deposition on the lens, and an early cataract and (b) showing presence of 360 degree posterior synechiae.

Synechiae may be present between the iris and the anterior lens capsule (posterior synechiae, PS) (Figures 3(a) and 3(b)) or may form between the peripheral iris and corneal endothelium as a result of previous iris bombe (peripheral anterior synechiae, PAS). When both are present, the PAS should be released before the PS. Release of PAS may be done by injecting viscoelastic, such as Healon 5 (Viscoadaptive from Abbott Medical Optics, Inc. Abbott Park, Ill, USA), to physically separate the iris from the cornea, failing which the tip of the viscoelastic cannula may be used to sweep the iris away from the peripheral cornea as the viscoelastic material is being injected into the angle of the anterior chamber. This should be done very gently and carefully, taking care not to detach Descemet's membrane in the process. PS may be lysed by simply injecting viscoelastic against the adherent iris, allowing the viscoelastic to “bulldoze” the iris away from the anterior capsule. Alternatively, this may be done by sweeping the pupil free from the lens capsule with a cannula. In cases where a narrow strip of membrane is present at the site of PS formation, a 27-gauge needle may be used to simultaneously nick the membrane to segment and release it from the anterior capsule and release the PS, thus stretching the pupil.

**(g) Pupil Expansion**

**Figure 4:**
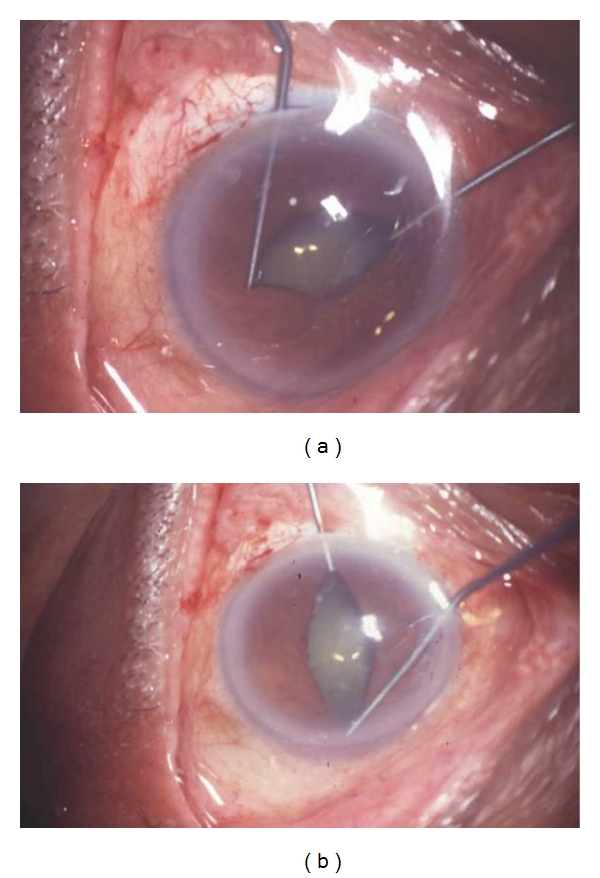
Intraoperative use of Kuglen hooks to stretch and dilate the pupils.

**Figure 5:**
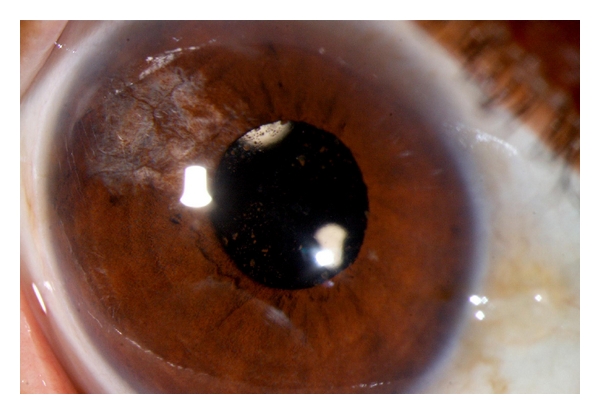
Postoperative slit lamp photograph showing minimally distorted pupil following pupil manipulation intraoperatively to negotiate posterior synechiae.

The most user-friendly instrument for extensive synechiae once an edge of the iris has been viscodissected off the anterior capsule is a bent Kuglen hook. This “push-pull” instrument is excellent for the safe release of PS, ranging from mild to extensive, as it enables the surgeon to push or pull the iris, thereby releasing the iris from the anterior lens capsule, even when a pupillary membrane is present. When the pupil has been freed, the membrane can be removed using a pair Kelman-Mcpherson forceps. Often, once the pupillary membrane has been removed, the pupil begins to widen with viscoelastic. However, if this is inadequate, the pupil may be stretched using a pair of angled Kuglen hooks introduced through the main incision, used in a manner to latch around the pupil edge, pulling the iris in opposite directions (Figure 4(a)). This is then repeated in a direction perpendicular to the initial stretch (Figure 4(b)). The surgeon should stop at once should the iris sphincter develop a tear. If proper execution of pupil manoeuvring is done, postoperatively pupil looks relatively round with minimal distortion of pupillary margins (Figure 5). The pupil may then be further enlarged using multiple sphincterotomies by means of intraocular scissors, taking care not to compromise the anterior capsule.

**Figure 6:**
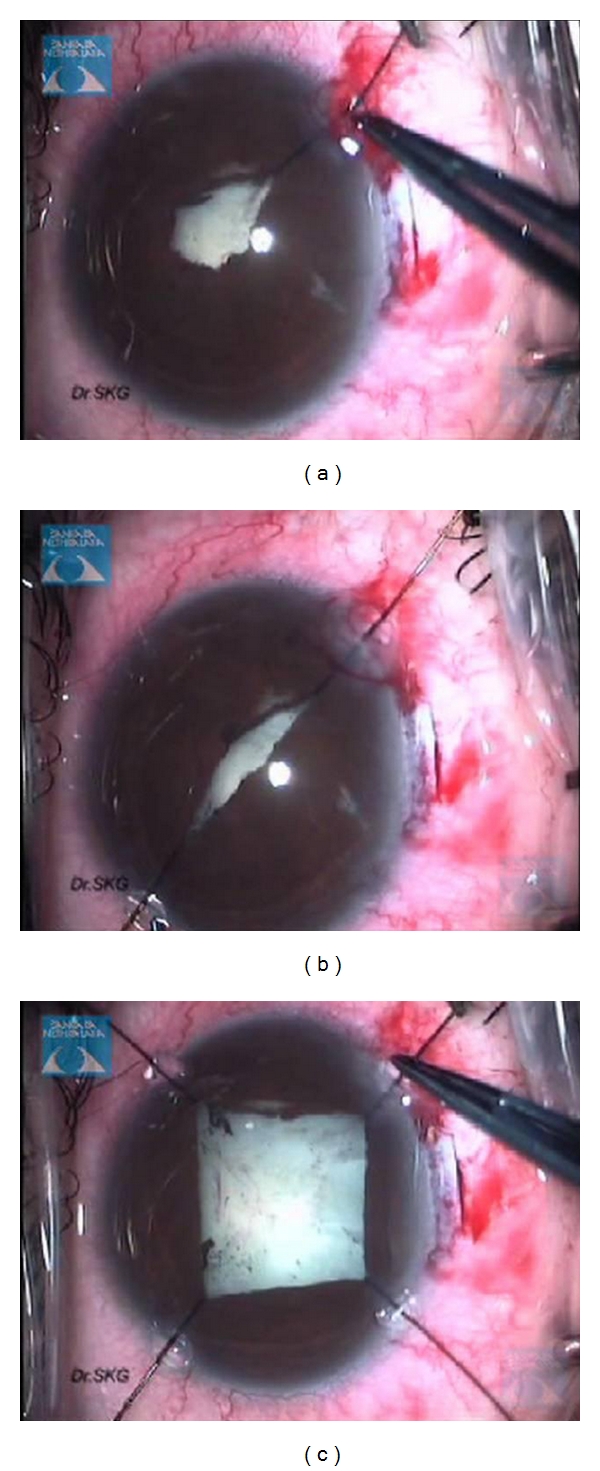
Intraoperative use of self-retaining iris hooks to dilate the pupils.

**Figure 7:**
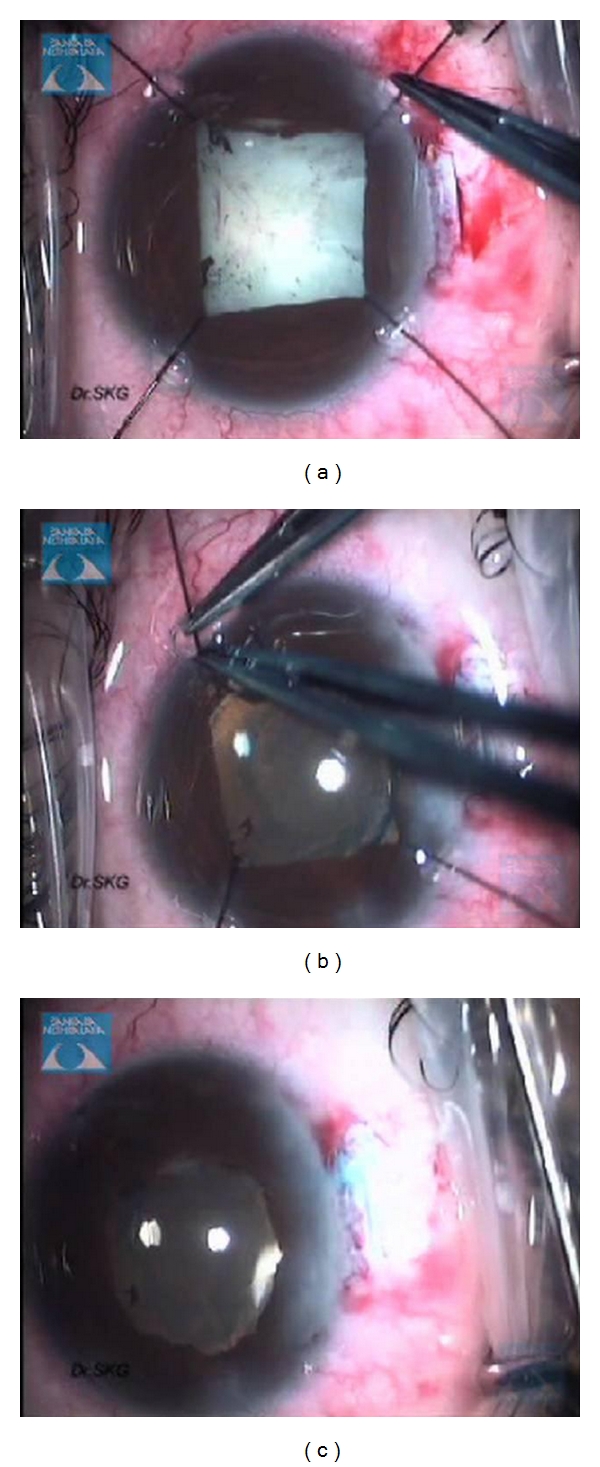
Postoperatively removal of self-retaining iris hooks.

Alternative means of opening the pupil include the use of a Beehler pupil dilator (2 or 3 pronged) to mechanically stretch the pupil in a single injector system. Pupil retainers may also be tried. Disposable iris hooks are easy to place through multiple corneal paracentesis (Figures 6(a)–6(c)). The iris hooks are removed at end of surgery (Figures 7(a)–7(c))). More recently a pupil device, the Malyugin ring (Microsurgical Technologies, Redmond, Wash, USA) has been used, which may be injected into the anterior chamber through a 2.2 mm incision and manoeuvred to expand and maintain the pupil open at a 6 or 7 mm diameter.

**(h) Continuous Circular Capsulorhexis**

**Figure 8:**
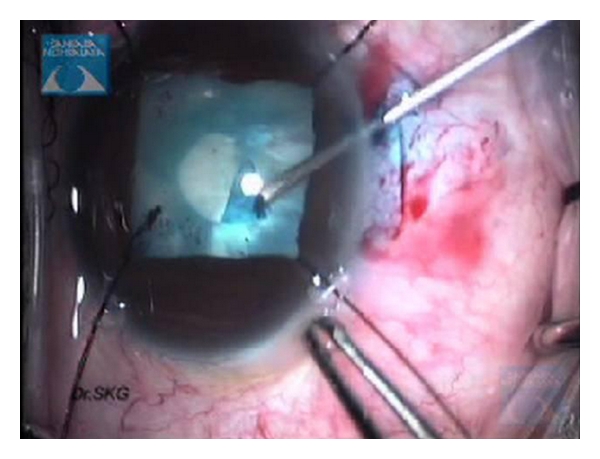
Intraoperative still video clip demonstrating the step of capsulorrhexis.

It is generally preferable to keep the size of the capsulorhexis slightly smaller than the pupil so that iris chaffing does not occur and results in progressive intraoperative miosis as nuclear fragments are being moved out of the capsular bag (Figure 8). This also contributes to increased postoperative inflammation. When the pupil size is small, the capsulorhexis inevitably needs to be larger than the pupil. The anterior chamber must be kept deep and the anterior capsule flattened using adequate viscoelastic material in order to control the tearing of a capsulorhexis. The capsulotomy can be initiated by a 26- or 27-gauge bent cystitome and a modified vitreoretinal forceps (pediatric rhexis forceps) can be then inserted from the side port to complete the rhexis. Creating the ideal capsulorhexis is also very important in preventing posterior capsule opacification. The capsulorhexis should be centred, overlapping the edge of the optic at all times, but not so small as to prevent capsular phimosis.

**(i) Nucleus Management**

**Figure 9:**
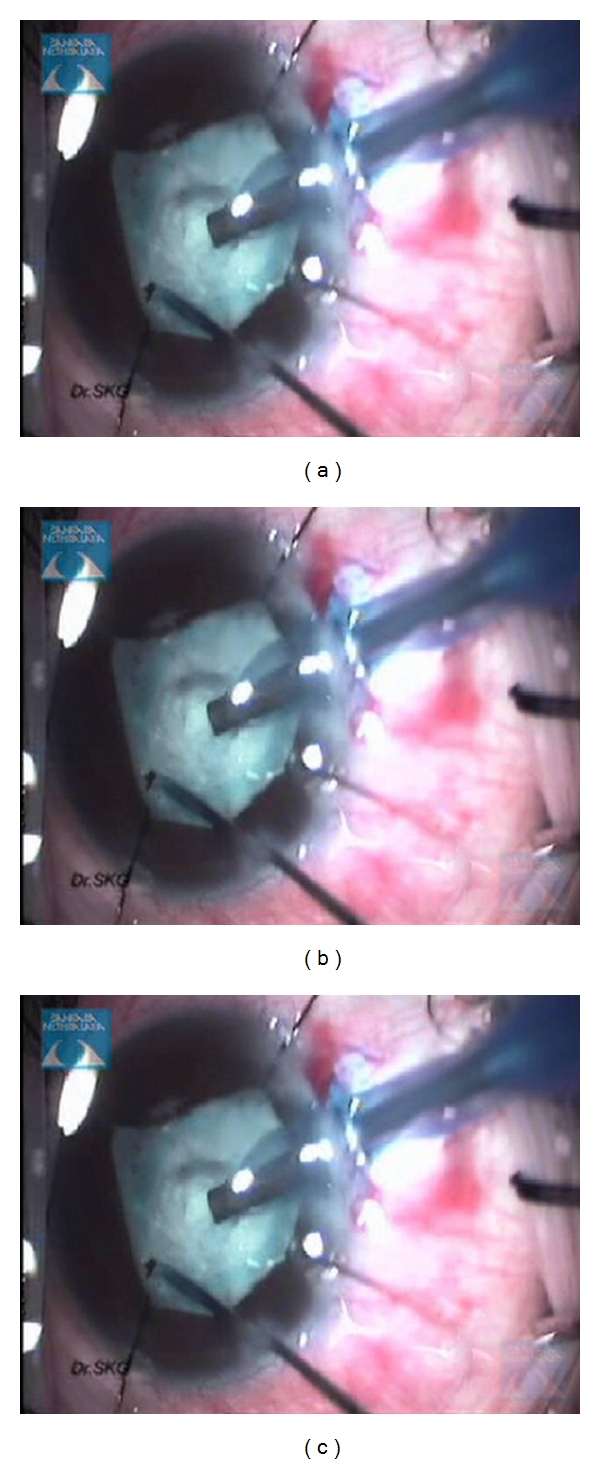
Intraoperative still surgical video clip showing the nucleus management in total white uveitic cataract.

In small pupils, the safest technique is the vertical chop employed in the in situ chop technique. Chopping of fragments is done within the pupillary aperture with the phaco tip kept in view at all times with minimal risk of engaging and traumatizing the iris (Figures 9(a) and 9(b)).

**(j) Irrigation and Aspiration**

**Figure 10:**
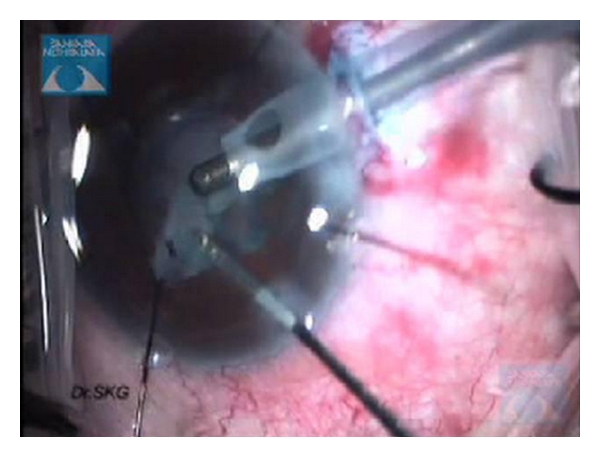
Intraoperative still surgical video clip showing irrigation and aspiration of the soft lens matter.

This step must be done thoroughly so as not to leave cortical material behind. The eye should be rotated to look for residual lens matter and a gentle shake given at the end of nucleus removal to ensure that no fragments are still lodged in the posterior chamber during phaco (Figure 10).

**(k) Intraocular Lens Implantation Contraindications and Type of IOL**

The review of the literature suggests that while earlier, implantation of IOLs in uveitic eyes with JIA and chronic uveitis was considered a contraindication, now with modern IOLs, it may be safe to implant IOLs in these difficult case scenarios as long as the uveitis is well under control. Alió et al. [39, 79] showed that the most biocompatible IOL for the anterior chamber and the capsular bag is a single-piece, square-edged acrylic (either hydrophilic or hydrophobic) IOL. They also found that the posterior capsular opacification rate was highest (34.2%) in eyes with silicone IOLs. In addition, silicone IOLs had a higher incidence of postoperative cystoid macular edema and PS formation, and pupillary membranes were formed only in eyes with silicone IOLs. In general, hydrophilic acrylic material has good uveal but worse capsular biocompatibility, but hydrophobic acrylic material had lower uveal but better capsular biocompatibility [78]. In eyes with chronic uncontrolled uveitis, IOL implant should be deferred.

Removal of viscoelastic from under the IOL is an important step in reducing the space behind the IOL into which lens epithelial cells tend to migrate, thus causing PCO. Pressing the optic against the posterior capsule when using a single-piece hydrophobic IOL also encourages its adhesion to the posterior capsule, thereby reducing the risk of PCO [79].

### 5.3. Complications and Postoperative Management

#### 5.3.1. Intraoperative Complications

**Zonulolysis**

An infrequent intraoperative complication is Zonulolysis. This can occur in eyes with chronic uveitis. Insertion of a plain capsular tension ring (CTR) is often necessary in order to prevent IOL decentration. However, if the overall zonular strength is weak, fixation of the CTR to the sclera by means of a modified Cionni CTR ensures that the IOL remains centred. Failure to use a CTR in the presence of weak zonules may result in capsular phimosis, due to unopposed capsular bag fibrosis and shrinkage.

**Retained Lens or Nuclear Fragments**

Due to the small pupil size, small hard nuclear fragments may lodge in the posterior chamber during phacoemulsification, only to pop into the anterior chamber months later. These small fragments can cause recurrent anterior uveitis when their position in the anterior chamber changes and may also cause localized corneal edema and even localized corneal decompensation in the long term. Hence, at the end of phaco, the eye should be given a gentle shake whilst aspirating to ensure no nuclear fragments are inadvertently left behind. Any retained soft lens material or nuclear fragment should be removed surgically as soon as possible [80].

#### 5.3.2. Early Postoperative Complications

**Excessive Postoperative Inflammation**

**Figure 11:**
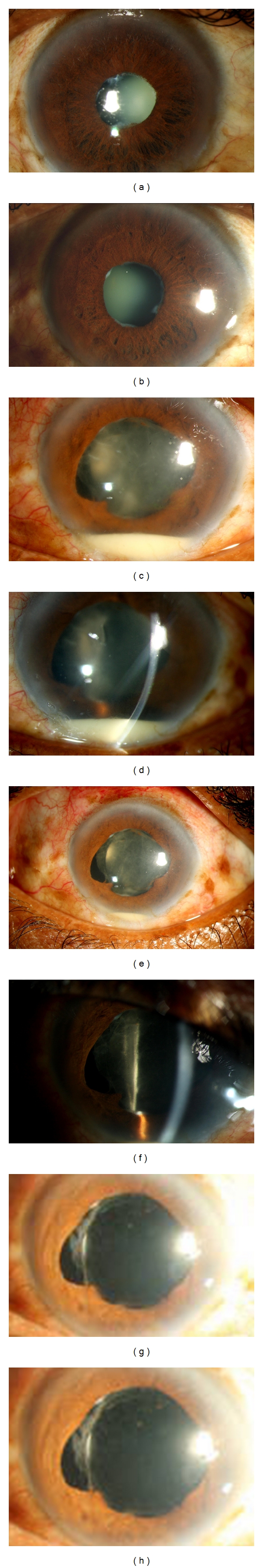
A 45-year-old man, a treated case of Hansen's disease 15 years ago, presented with progressive visual loss. Examination showed active anterior uveitis and complicated cataract. He was treated with topical steroids and after 4 months, underwent phacoemulsification with PCIOL in the left eye. On the first postoperative day, his visual acuity improved to 20/50 (from preoperative vision of 20/200) with a mild fibrinous reaction in the anterior chamber. He returned to the emergency clinic on the next day with loss of vision and showed severe anterior chamber reaction with hypopyon. Vitreous appeared uninvolved. He was hospitalized and treated with intensive topical steroids and cycloplegics. He improved over the course of one week and regained good vision at the end of one month. (a, b) show the right and left eye with quiet anterior chambers and nondilating pupil with posterior synechia. (c, d) Diffuse and slit view of the left eye on the second postoperative day shows hypopyon and coagulum around the IOL. (e, f, g) Diffuse low and high magnification and slit view of the left eye two days after intensive treatment showing decrease in the inflammation. (h) The left eye shows near-quiet anterior chamber 2 weeks after treatment, the visual acuity has improved to 20/50.

One of the most common postoperative complications is excessive postoperative inflammation (Figures 11(a)–11(g)). This may vary in terms of severity or duration of inflammation. Associated with this is the development of cystoid macular edema, which may be treated by controlling the inflammation. The incidence of CME following extracapsular cataract surgery in uveitic eyes is frequent and has been reported to range from 33% to 56% [10, 14]. Following phacoemulsification, the incidence has been reported to range from 12% to 59% [38]. Generally, if preoperative prophylactic oral steroids have been given and maximal topical steroids and cycloplegics have proven ineffective in controlling the uveitis, the dose of oral steroids may be sharply increased. If no prophylactic oral steroids had been given the patient should be given an oral pulse of steroids or injection of periocular steroids. An alternative means would be to give an intravitreal injection of triamcinolone acetonide [81] if this had not been given intraoperatively, thus avoiding the need to adjust the systemic immunosuppression. In the pediatric eye with chronic uveitis undergoing cataract surgery, a multistage surgery may be the safer approach, whereby various complications are addressed at different sittings [79]. This strategy may avoid postoperative complications and improve surgical outcomes.

**Figure 12:**
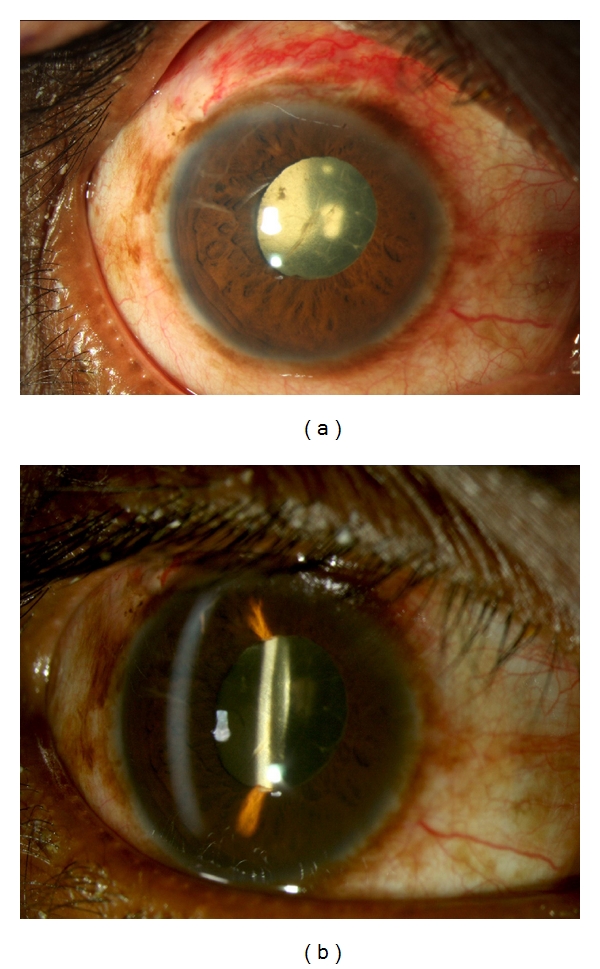
A 36-year-old man presented with a history of redness and pain since two years and decreased vision since one year. Examination showed a total cataract in the right eye, with 360 degrees posterior synechiae. Investigations showed HLA-B 27 was positive. After the inflammation subsided, the patient underwent cataract surgery with synechiolysis. Postoperatively, there was increased anterior chamber inflammation, which was treated with oral and topical steroids. The patient regained visual acuity of 20/25; 3 months postoperatively and is on maintenance with oral methotrexate and topical steroids. (a, b) Diffuse and slit photograph of the right eye on the first postoperative day shows a membrane on the IOL which responded to intensive topical steroids and cycloplegics.

Posterior synechiae, pupillary or ciliary membrane formation may occur during the postoperative period due to excessive inflammation (Figures 12(a) and 12(b)). Control of uveitis and keeping the pupil mobile during this time are important.

**Intraocular Pressure (IOP) Abnormalities**

The IOP may be raised transiently during the early postoperative period in eyes with compromised trabecular meshwork or angles. This can often be managed with topical and systemic anti-glaucoma medications.

However, the surgeon's greatest fear is hypotony. Once wound leakage has been ruled out, the next step is to increase the anti-inflammatory therapy topically and systemically. This is often effective in raising the IOP, but the topical steroids may be difficult to taper or withdraw and patients may require long-term topical steroids to maintain the IOP. Stabilisation of the IOP and vision has been successfully treated with an intraocular injection of sodium hyaluronate via a limbal paracentesis in non uveitic eyes [82]. In severe cases, vitrectomy and trimming of ciliary body traction membranes and silicone oil filling may be needed if UBM shows the presence of ciliary body detachment secondary to tractional membranes not addressed during the cataract surgery [83].

**Recurrence of Uveitis**

Increased frequency of recurrence following cataract surgery may occur. This is thought to be triggered by the intraocular procedure. The recurrence rate has been reported to be as high as 51% [15]. Hence, stepping up the immunosuppression for the long term may be necessary to prevent further recurrences.

#### 5.3.3. Late Postoperative Complications

**Posterior Capsular Opacification**

In the late postoperative period, posterior capsular opacification is perhaps the most common complication following any type of cataract surgery. Okhravi et al. [9] reported an incidence of 48.0%, Rauz et al. [84] 81.7% and Küçükerdönmez et al. [82] 34.2% at 1 year. Their corresponding Nd:YAG capsulotomy rates were 32.2%, 8.3%, and 3.6%.

Preventive measures include creating a circular well-centred capsulorhexis which is smaller than the optic size, using an acrylic IOL with a square-edged optic design, meticulous removal of viscoelastic from within the capsular bag and ensuring the optic is stuck on to the posterior capsule at the conclusion of surgery. Control of postoperative inflammation also plays an important role in preventing PCO.

**Explanting an IOL**

Removal of intraocular lens in uveitic eyes is rarely necessary. Foster et al. reported that their indications include the formation of perilental membrane, chronic low-grade inflammation not responding to anti-inflammatory treatment and cyclitic membrane resulting in hypotony and maculopathy [85, 86]. The underlying diagnosis for uveitis included sarcoidosis, JIA, and pars planitis, in eyes with predominantly intermediate or panuveitis, with the inflammation centered on the pars plana region. They believe that undetected subclinical inflammation present chronically after surgery was responsible for the postoperative complications leading to IOL removal despite the necessary precautions having been taken during the perioperative period.

## 6. Conclusion and Summary

 It is possible to achieve successful visual outcomes following cataract surgery in uveitis with the modern day cataract surgery. The predictability has improved mainly because of a higher level of understanding of the uveitic disease among clinicians. Preoperative factors include proper patient selection and counseling and preoperative control of inflammation. It is now well recognized that chronic inflammation, even low grade, can irreversibly damage the retina and optic nerve [6], and therefore control of inflammation, both pre- and postoperatively, is vital. The use of immunosuppressive agents other than steroids also helps control inflammation and has enabled long-term use of these agents especially as steroids sparing medication. Management of postoperative complications, especially inflammation and glaucoma, earlier rather than later, has also contributed to improved outcomes. Still several questions remain unanswered, especially in the area of pediatric uveitis with cataract, which continue to challenge the ophthalmologist to further refine the surgical technique and search for new treatment modalities [56, 57]. In conclusion, management of the uveitic cataract requires careful case selection, proper timing of surgery, meticulous surgery and close monitoring with appropriate handling of the postoperative complications that may occur. These eyes can achieve good outcomes with proper management.
